# Comparing Tactile to Auditory Guidance for Blind Individuals

**DOI:** 10.3389/fnhum.2019.00443

**Published:** 2019-12-19

**Authors:** Arnav Bharadwaj, Saurabh Bhaskar Shaw, Daniel Goldreich

**Affiliations:** ^1^Department of Psychology, Neuroscience & Behaviour, McMaster University, Hamilton, ON, Canada; ^2^McMaster Neuroscience Graduate Program, McMaster University, Hamilton, ON, Canada

**Keywords:** visual impairment, waypoint, navigation, haptic, vibration, blindness, spatial orientation and wayfinding

## Abstract

The ability to travel independently is crucial to an individual’s quality of life but compromised by visual impairment. Several navigational aids have been developed for blind people to address this limitation. These devices typically employ auditory instructions to guide users to desired waypoints. Unfortunately, auditory instructions may interfere with users’ awareness of environmental sounds that signal dangers or provide cues for spatial orientation. Accordingly, there is a need to explore the use of non-auditory modalities to convey information for safe and independent travel. Here, we explored the efficacy of a tactile navigational aid that provides turn signals *via* vibrations on a hip-worn belt. We compared the performance of 12 blind participants as they navigated a series of paths under the direction of the tactile belt or conventional auditory turn commands; furthermore, we assessed the effect of repeated testing, both in the presence and absence of simulated street sounds. A computer-controlled system triggered each turn command, measured participants’ time-to-path-completion, and detected major navigational errors. When participants navigated in a silent environment, they performed somewhat worse with the tactile belt than the auditory device, taking longer to complete each trial and committing more errors. When participants navigated in the presence of simulated street noises, the difference in completion time between auditory and tactile navigation diminished. These results suggest that tactile navigation holds promise as an effective method in everyday environments characterized by ambient noise such as street sounds.

## Introduction

In order to navigate safely and efficiently, blind individuals attend to nonvisual environmental stimuli, such as street sounds, while making use of mobility aids. Common mobility aids include the white cane and guide dog, which provide information about the user’s immediate surroundings, and Global Positioning System (GPS) devices, which provide location and heading information. Despite the usefulness of these aids, much research is needed to develop more effective navigational devices for blind individuals (Loomis et al., 1994; Giudice and Legge, 2008).

Assistive navigational devices for blind people typically employ auditory instructions to guide users (Loomis et al., 2005; Gaunet, 2006). Despite their obvious usefulness, these instructions may prevent users from perceiving simultaneous environmental sounds that signal dangers or provide cues for spatial orientation—for instance, sounds made by passing cars, by nearby pedestrians, or by other sources. The presence of two simultaneous sources of auditory information places demands on attentional processing and raises the possibility of physical acoustic interference. Imagine navigating without vision in the midst of a busy, unfamiliar environment. A “turn left” auditory GPS command might interfere with the sound of a car driving by, with potentially lethal consequences. Alternatively, a car may be honking nearby, such that you’re unable to decipher the auditory commands emanating from your GPS device. When two or more sound sources are simultaneously active, distinguishing them is difficult, as their acoustic waveforms sum into a composite waveform prior to entering the ear (Bregman, 1990; Darwin, 1997).

In light of these considerations, the most suitable navigational aid in acoustically rich environments may be one based upon the sense of touch. Conveying navigational commands *via* touch would decouple the two sources of information—navigational commands and environmental sounds—preventing both physical and attentional interference between them. The present study was designed to test this proposition and to investigate blind individuals’ ability to process simple navigational instructions *via* the skin.

The somatosensory system would seem to offer a reliable communication channel for navigational purposes. The skin has a large available surface area; the point-to-point mapping from the skin to the somatosensory homunculus naturally conveys spatial information (Nakamura et al., 1998); and vibrations applied in sequence to adjacent skin locations can be accurately interpreted as directional information (Raj et al., 1998; Chiasson et al., 2002; Cholewiak et al., 2004; Van Erp et al., 2005; Jones and Ray, 2008; Barber et al., 2015).

In light of these promising characteristics, tactile-directed navigation has been a focus of research and development for many years (Bach-y-Rita, 1967; Ertan et al., 1998; Tsukada and Yasumura, 2004; Van Erp et al., 2005; Johnson and Higgins, 2006; Gustafson-Pearce et al., 2007; Pielot and Boll, 2010; Flores et al., 2015; Jimenez and Jimenez, 2017). Ertan et al. (1998) successfully guided sighted participants through indoor test paths by passing directional commands to a wearable vest containing an array of vibrotactile actuators. Similarly, Tsukada and Yasumura (2004) developed the “ActiveBelt” to guide participants to waypoint destinations, with tactile commands based on GPS. In order to assess the efficacy of tactile displays, Van Erp et al. (2005) and Pielot and Boll (2010) compared participants’ navigational performance with a tactile display to that with a visual display. Both studies showed promising results for the potential use of tactile displays as hands-free guidance systems. In a study, with standing, stationary participants, Gustafson-Pearce et al. (2007) demonstrated that visually impaired and sighted participants could accurately follow tactile turn commands delivered through a vest; they further showed that, in the presence of simulated street sounds, participants made fewer errors in response to tactile than to auditory commands.

Most recently, Flores et al. (2015) and Jimenez and Jimenez (2017) compared navigation performance with a tactile display to that with an auditory device. In a study with blind participants, Flores et al. (2015) used an automated participant localization system to precisely transmit directional commands to both navigational devices. They found that navigation was slower but more accurate in the tactile condition. Jimenez and Jimenez (2017) transmitted either auditory or vibrotactile navigational commands to blindfolded sighted participants. They found that navigation was slower and more error-prone in the tactile condition. While both these studies found that navigation was slower when directed *via* tactile displays, neither study assessed improvement in performance with repeated testing or how performance was affected by conditions of high sensory load. We wondered whether tactile navigation, as it is less familiar to users, might be slower initially but become more efficient with practice.

Here, we extend this body of research. We compared the performance of blind participants as they navigated under the direction of auditory commands or tactile belt commands. Additionally, we assessed the effect of repeated testing in a controlled environment, in the presence or absence of simulated street sounds. For the reasons outlined above, we predicted that: (1) in the absence of street sounds and with sufficient practice, tactile navigation would be at least as effective as auditory navigation; and (2) in the presence of street sounds, tactile navigation would be superior to auditory navigation. The study’s results, while intriguing, corresponded only partially to these predictions.

## Materials and Methods

### Participants

We conducted experiments with 14 blind adults (11 men and three women, ranging in age from 21 to 60 years; mean, 39.9 years). Exclusion criteria ensured that blind participants did not have impairments known to affect tactile sensation, that blindness was of peripheral origin, that the participants’ degree of vision did not exceed residual light perception (ability to perceive light but not form), and that no participant had diabetes, hearing problems, balance difficulties, tremor, epilepsy, multiple sclerosis, stroke, neurological disorders, learning disabilities, dyslexia, attention deficit disorders or cognitive impairments. All participants gave signed consent (consent form read aloud by an investigator) and received monetary compensation for their participation. All procedures were approved by the McMaster University Research Ethics Board.

The participants had no more than residual light perception, but their visual histories were quite varied. At one extreme were participants born with normal vision who then progressed through a stage of low vision (defined here as the ability to perceive both light and form) to reach residual light perception (perception of light but not form). At the other extreme were participants born with residual light perception or less. Defining childhood as the period between birth and 12 years of age, we classified four participants as congenitally blind (residual light perception or less at birth), two as early-blind (normal or low vision at birth declining to residual light perception or less by the end of childhood), and eight as late blind (normal or low vision throughout childhood, declining to residual light perception or less in adulthood). Nine participants had residual light perception at the time of testing and five had no light perception. Two participants were excluded from the analysis due to incomplete data as they were unable to complete the full experiment. Characteristics of the 12 included participants are summarized in Table 1.

**Table 1:** Participant characteristics.

	Participant Characteristics			
	Congenitally blind	Early blind	Late blind	Total
No vision	0	2	2	4
Residual light perception	3	0	5	8
			Total	12

### Equipment

**Figure 1:**
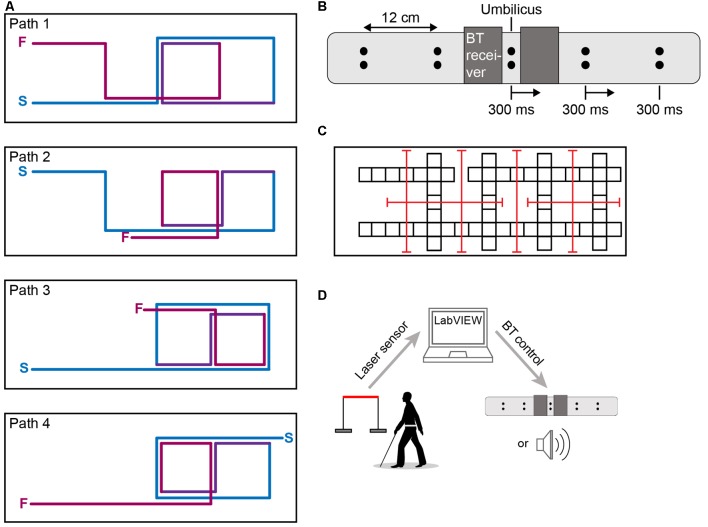
Apparatus. **(A)** Four walking paths equated for difficulty (10 turns, ~127 ft in length; S = path start; F = path finish). Lines slightly offset and colored for clarity only. **(B)** Vibrotactile navigational belt schematic. Each dot represents a coin motor. Vibrotactile stimulation began below the umbilicus and moved towards the left or the right (a rightward movement is shown for illustration). **(C)** Room layout. Each square represents a 2 ft. × 2 ft. mat. Red lines show the participant localization laser transmitter-receiver grid. **(D)** Systems overview. Image of person walking with cane is modified from ID 45773820 © Anastasia Popova ∣ Dreamstime.com.

The experiment was conducted in a 16.5 ft. wide by 51 ft. long room in the Psychology Building on the campus of McMaster University. Sturdy foam mats (2 ft × 2 ft) were arranged throughout the room to delineate walkways. Four distinct walking paths were defined and equated for difficulty, each containing 10 (90°) turns with similar lengths (one path was 127 ft and the rest were 128 ft; Figure 1A). The equivalent difficulty of these paths was confirmed by the similar times required by participants to complete them (one-way ANOVA on completion time: *F* = 0.249, *p* = 0.862).

Following the consent procedures, the tactile belt was fitted to the participant. The belt attached to the torso with the help of Velcro straps. It contained ten vibratory coin motors, each of diameter 10 mm, arranged in pairs at regular intervals (Figure 1B). The belt had elastic segments that allowed it to fit each participant comfortably and rigid segments that allowed for proper anchoring of the circuitry and wiring; due to the belt’s elasticity, the spacing between the motors scaled with the participant’s waist size. The coin motors, similar to those in smartphones, vibrated by spinning an imbalanced mass at a high speed. The peak-to-peak displacement of the vibration produced by the motors was 1.5 mm, at a frequency of 55 Hz. The direction of the sweeping vibration was controlled by LabVIEW 2014 on a Windows PC and communicated to the belt using a Bluetooth 4.0. Once a control was initiated, motors centered on the participant’s midline immediately began vibrating to cue participants for an upcoming turn. The motors subsequently vibrated consecutively at adjacent positions (0.3 s per location, 0 s ISI) in order to create a directional signal. Depending on the direction fed by the Bluetooth control, the vibration traveled from either midline to left or midline to right (Figure 1B). At the beginning of every tactile trial, the vibration traveled around the participant’s torso twice to prompt the participant to begin the task.

Attached to the same belt were two small Bluetooth audio speakers. The smaller, circular speaker (diameter 8 cm × thickness 3.5 cm) was attached to the front of the belt on the participant’s midline. Similar to the tactile belt, this speaker output navigational—“turn left” or “turn right”—female speech commands corresponding to the Bluetooth controls sent from the same LabVIEW program. To enable direct comparison between the command types, the duration of the auditory commands was also 1 s, and the “turn” portion of the auditory commands was analogous to the midline tactile vibration of the belt. Additionally, this speaker output a “please start” command at the beginning of every auditory trial to prompt the participant to begin.

The second, rectangular, speaker (14.5 × 7 × 2 cm) was attached to the back of the belt, behind the participant. Its function was to output background street noise, which was broadcast from an Android-device *via* Bluetooth. The street noise was played from this speaker mounted on the back of the belt, rather than a fixed speaker in the room because the use of a fixed speaker would provide spatial cues that could artificially facilitate the participant’s learning of the path. The streets sound recording played on continuous run during a trial and was not synchronized in any way to the location of the participant or to the turn commands emitted from the front speaker. The full sound recording was of approximately 14.5 min duration; the recording was stopped at the end of each trial and restarted, where it left off, at the beginning of the next trial. The recording consisted of various sound events (e.g., conversation, car horn, a truck backing up, dog barking, car skidding). The average sound event duration was 7.3 s (SD 5.3 s). Events could overlap in time (e.g., the sounds of children playing might overlap with the sound of bicycle bells or adults speaking). During the full 14.5 min recording, 50 silent intervals were interspersed between sound intervals. The mean silent interval was 7.9 s (SD 4.4 s).

Sound pressure level (dB SPL) was measured with an i436 omnidirectional professional microphone (MicW) using SoundMeter X, v 10.1 running on an iPhone 6 s. With the microphone placed approximately 80 cm above the speaker to simulate the distance from a participant’s belt to ears, the overall max level in repeated measurements averaged 76.1 dB for the street sounds, 77.5 dB for the turn left command, and 71.8 dB for the turn right command.

The participant localization system consisted of a grid of 6 laser beams distributed throughout the room (Figure 1C). The laser beams (5 mW, 650 nm, 20 mA red laser diodes) traveled parallel to the floor at a height of approximately 1 meter and passed through the participant’s walking path at a distance of 3 feet prior to an edge of a mat connected to an intersection. Sensors (CDS Cell 690 nm 0.17 ~2 kOhm @ 21 lux) detected the moment each beam was broken by the participant and relayed the change in voltage to the LabVIEW program *via* an NI USB-6008 I/O board. Upon receipt of the voltage signal, LabVIEW issued the Bluetooth signals to either the front speaker or belt, as appropriate to the experimental condition (system overview; Figure 1D). Due to hardware latencies, the time delays between beam break and auditory command onset, and beam break and tactile vibration command onset, were 298 ± 5 ms and 153 ± 34 ms, respectively.

The LabVIEW program recorded the times taken to successfully complete each path and the errors made during waypoint navigation (i.e., any instances during which the participant missed a turn, turned in the wrong direction, or walked off the course in any way). In the event of an error, the participant was told to stop walking and was returned to the start of the path to begin again. Additionally, the navigational behavior of participants was recorded by two cameras connected to a separate Windows 8 based PC.

### Navigational Testing

The testing consisted of four conditions in a 2 [navigational command type: tactile (T) vs. auditory (A)] by 2 [ambient noise: quiet (Q) vs. background street sounds (S)] repeated-measures experimental design (Table 2). This design allowed us to assess the efficacy of navigation with tactile commands compared to conventional auditory commands, under conditions of either quiet or background street sounds. Participants were given a 20-min practice phase prior to the commencement of testing. During practice, they had the opportunity to become familiar with the tactile belt and auditory device and the foam mats that made up the paths and to gain a basic understanding of the navigational task. They did so by navigating through a practice path that was significantly different and shorter than the test paths. During the practice and all testing sessions, investigators stood silently at strategic locations within the room, in order to intervene if necessary (i.e., to tell participants to stop walking if they were off-path and in danger of colliding with a wall or other object).

**Table 2:** Testing conditions.

	Tactile belt (T)	Auditory device (A)
Quiet (Q)	TQ, 5 Trials: Time and error	AQ, 5 Trials: Time and error
Sound (S)	TS, 5 Trials: Time and error, comprehension	AS, 5 Trials: Time and error, comprehension

For all participants, Quiet testing with both navigational command types preceded Sound testing. This was done for the safety of the participants, such that the participants faced less difficult tasks initially and would not be distracted from hearing the voices of the investigators, should it be necessary for safety reasons for the investigators to intervene. Half of the participants completed conditions in the order A-Q, T-Q, A-S, T-S; the other half completed conditions in the order T-Q, A-Q, T-S, A-S (Table 3). Participants were required to successfully complete five trials in each condition before proceeding to the next condition. Each condition used a different path from among four designed paths. The paths corresponding to the four conditions were counterbalanced across participants so that all paths were equally used for each condition. Participants were required to take a minimum 2-min rest after each trial within a condition, and a 5-min rest period between conditions.

**Table 3:** Order of testing by participant.

	Order of testing (Condition-Path) by participant			
Participant	Testing order			
	1	2	3	4
1	AQ-1	TQ-2	AS-3	TS-4
2	TQ-1	AQ-2	TS-3	AS-4
3	TQ-2	AQ-1	TS-3	AS-4
4	AQ-2	TQ-1	AS-3	TS-4
5	TQ-1	AQ-2	TS-4	AS-3
6	AQ-1	TQ-2	AS-4	TS-3
7	TQ-2	AQ-1	TS-4	AS-3
8	AQ-2	TQ-1	AS-4	TS-3
9	TQ-3	AQ-4	TS-1	AS-2
10	AQ-3	TQ-4	AS-1	TS-2
11	TQ-3	AQ-4	TS-2	AS-1
12	AQ-3	TQ-4	AS-2	TS-1

Participants were instructed to walk at their normal, comfortable speed and to use their white cane as they typically would while navigating a path. They were instructed to walk straight along a path until receiving a turn command, and to make 90° left or right turns upon receipt of the corresponding left or right command. Participants were told to try to avoid errors (i.e., stepping off the path or making a wrong turn). Except for the mode of command (vibrations delivered by the belt or auditory instructions issued *via* a belt-attached portable speaker), the protocol was identical in the tactile and auditory command conditions.

At the end of each trial in a Sound condition, a co-investigator questioned the participant as to what street sounds (s)he heard within the timeframe of the last trial. All recall questionnaires were identical, asking if the participant heard a specific event. Participants were required to choose from “yes,” “no,” or “unsure” for every question. On any particular trial, sounds may have included dogs barking, car horns, large truck back-up beeps, ambulance/police sirens, bike bells, and/or people talking.

At the end of the experiment, the participant was asked to respond to a series of follow-up questions regarding their experience with the tactile system in comparison to the auditory commands, in order to provide ideas for the future development of the device.

### Navigational Data Collection and Analysis

During navigation, the time at which the participant broke each laser beam was automatically recorded by the computer program. Major navigational errors were defined as failing to respond to navigational instructions or making wrong turns. These errors were picked up by the beam/sensor system and automatically recorded by the computer. Minor navigational errors were defined as stepping off the path such that more than half a foot was off the path. These errors were recorded by two co-investigators who were situated in the corners of the room and observed the participant visually. The number of minor errors recorded by the two co-investigators was averaged if the tally differed among the co-investigators. If the difference was large, the video recording of the corresponding trial was viewed to determine the correct number of minor errors. The path completion time was recorded by the computer as the time elapsed between the first and final beam breaks.

We performed repeated-measures ANOVAs with type III sum of squares and two-tailed *t*-tests using SPSS Statistics version 25 (IBM) for Windows with an alpha level of 0.05, in order to assess the effects on the dependent measures of command type (i.e., Tactile vs. Auditory), ambient noise (Quiet vs. Sound), and repeated testing (i.e., trial number). For the purpose of the three-way repeated-measures ANOVA on minor errors, if a particular trial number was terminated due to a major error and consequently re-run one or more times, we averaged the number of minor errors from the runs.

## Results

We assessed the ability of blind participants to navigate paths using either tactile or auditory commands and in the absence or presence of background street sounds. We measured navigational performance as the time taken to complete each trial and the number of errors committed.

### Participants Committed More Major but Not Minor Errors When Using the Tactile Belt

All 12 participants completed the five trials per condition successfully within the allotted experimental time. However, the navigation task was somewhat challenging, as indicated by the observation that every participant had at least one major error (i.e., missed turns or wrong turns); consequently, every participant was required to repeat a trial at least once. The mean number (± SE) of major errors produced by the participants across all conditions was 4.0 ± 0.8. At the extremes, P5 and P11 committed only one error each, whereas P3 and P6 committed eight errors each. For minor errors, the mean was 29.8 ± 4.1, with the extremes being P7 with three errors and P12 with 51 errors.

The average number of major and minor errors committed per participant in each testing condition is shown in Table 4. Participants committed more major errors under tactile than auditory commands, in both the Quiet and Sound conditions. Additionally, participants tended to commit more major errors, within each command type condition, with the addition of background street sound. Nevertheless, a two-way (command type × ambient noise) repeated-measures ANOVA on committed major errors indicated a significant effect of command type (*F* = 5.046, *p* = 0.046) only, with no significant effect of ambient noise (*F* = 1.244, *p* = 0.288). We did not analyze the effect of trial number on major errors, as the number of major errors committed was too small to support such an analysis.

**Table 4:** Major and minor errors.

Major and minor errors (Mean ± SE) by condition averaged across participants			
Ambient noise	Command type	Major errors	Minor errors
Quiet (Q)	Tactile (T)	1.25 ± 0.46	7.88 ± 1.54
	Auditory (A)	0.33 ± 0.22	8.46 ± 1.49
Sound (S)	Tactile (T)	1.58 ± 0.43	6.96 ± 1.05
	Auditory (A)	0.83 ± 0.37	6.50 ± 1.15

In contrast to the result with major errors, the number of minor errors did not differ significantly between the two command types. A 2 × 2 × 5 (command type × ambient noise × trial) three-way repeated-measure ANOVA on minor errors indicated no effect of command type (*F* = 0.203, *p* = 0.660) or trial (*F* = 0.224, *p* = 0.924) and no significant two-way interactions. Participants committed significantly fewer minor errors in the street sound condition (*F* = 6.943, *p* = 0.022), perhaps reflecting an effect of practice, as the Sound condition occurred in the second half of the experiment.

In summary, participants committed fewer major but not minor errors when using auditory navigational commands, and performance did not significantly worsen with the addition of background street sounds.

### Recall Performance Was Equivalent for the Two Command Types

We next compared the ability of participants to recall events from the background street sounds (Sound conditions) while navigating with either device. Most participants performed well on the recall questionnaire signifying that they were actively attending to the background street noise. A two-way (command type × trial) repeated-measures ANOVA verified that there was no significant effect of either command type or trial (*F* = 0.441, *p* = 0.520; *F* = 0.224, *p* = 0.923) on the number of correct responses. These results indicate that participant recall was equivalent across navigational devices, signifying that participants were equally and actively attending to the background street noise while navigating with both devices. Additionally, participants’ ability to recall events from their immediate environment did not change with practice or increasing number of trials.

### Improvement With Practice Was Statistically Similar in Auditory and Tactile Navigation

**Figure 2:**
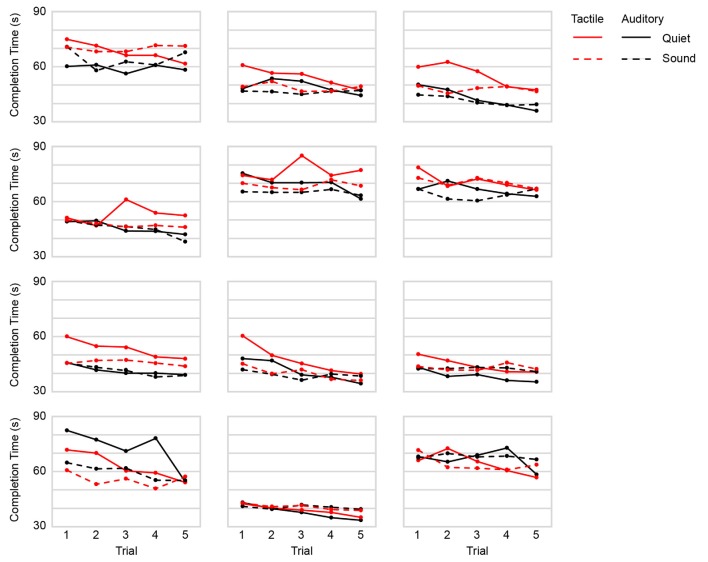
Completion times as a function of trial by condition. Panels: individual plots of all participants (*n* = 12). Red: tactile navigational commands. Black: auditory navigational commands. Solid: quiet. Dashed: street sound.

The time taken by the participants to complete each of the five navigational trials in the four conditions is shown in Figure 2. Completion times consistently diminished as a function of trial number, indicating that participants improved with practice in every condition.

**Figure 3:**
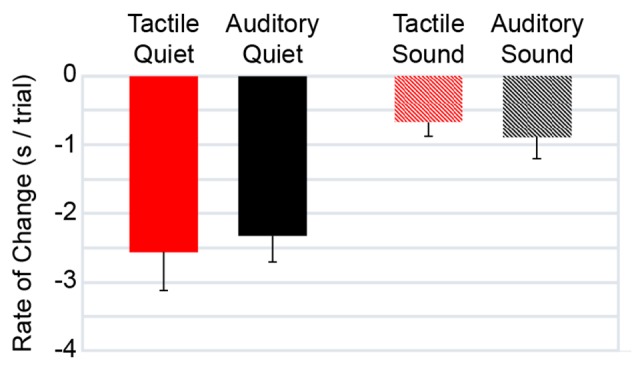
Rate of change in completion time by condition. Bars: mean regression slopes derived from completion times as a function of trial (Note: negative slopes indicate improvement—i.e., reduction—in completion time). Red: tactile navigational commands. Black: auditory navigational commands. Solid: quiet. Hatched: street sound. Errors bars: 1 SE.

For each participant and each condition, we determined the best-fit line relating completion time to trial number by linear regression; the slopes of these best-fit lines indicate the improvement in completion time with practice (Figure 3). We investigated whether the slopes differed across conditions. A two-way (command type × ambient noise) repeated-measures ANOVA on slope revealed a significant effect of ambient noise (*F* = 23.886, *p* < 0.001) with no significant effect of command type (*F* = 0.008, *p* = 0.931) and no significant ambient noise × command type interaction (*F* = 0.615, *p* = 0.450). The slopes in the Quiet conditions were steeper than in the Sound conditions, indicating greater improvement with repeated testing in the Quiet conditions. This difference in the rate of improvement may have occurred because the Quiet conditions came first in the experiment. The non-significant effect of command type indicates a similar rate of navigational improvement with the two devices.

### In the Absence of Ambient Noise, Auditory Navigation Was Faster Than Tactile Navigation

**Figure 4:**
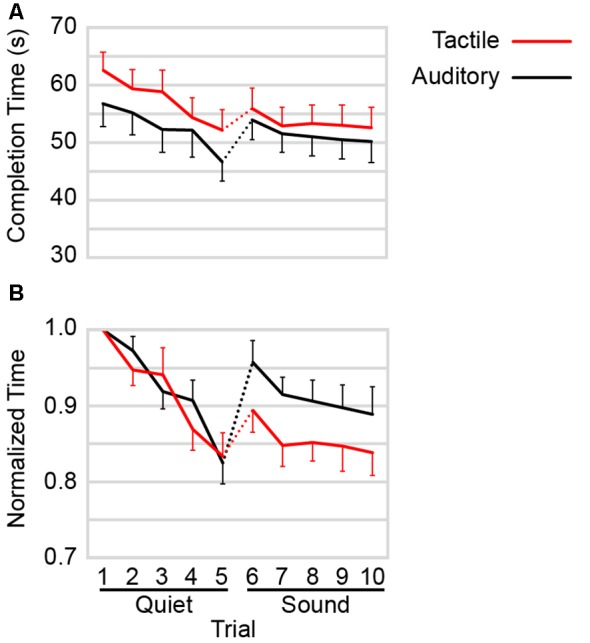
**(A)** Mean completion time as a function of trial by command type. Data from Figure 2 were replotted by averaging across all participants (*n* = 12) for each condition. **(B)** Mean of completion times normalized against trial 1. For each participant, completion time on each trial was divided by the completion time from trial 1 of the corresponding command type. Red: tactile navigational commands. Black: auditory navigational commands. Dotted lines: background street sounds introduced into the experiment. For visual clarity, error bars show +1 SE and −1 SE, respectively for the highest and lowest points at each comparison distance.

The participants’ mean completion times for each trial of each condition are shown in Figure 4A. A 2 × 2 × 5 (command type × ambient noise × trial) three-way repeated-measure ANOVA on the completion times revealed a highly significant effect of trial (*F* = 28.639, *p* < 0.001), a significant effect of command type (*F* = 6.678, *p* = 0.025), and a significant effect of ambient noise (*F* = 5.066, *p* = 0.046). The ANOVA further revealed a significant command type × ambient noise interaction (*F* = 7.004, *p* = 0.023).

Figure 4A suggests that the significant interaction was due to a larger difference in completion times between auditory and tactile command types in Quiet than in Sound. To investigate this, we conducted two separate *post hoc* two-way (command type × trial) repeated-measures ANOVAs, one for Quiet and one for Sound. These ANOVAs revealed a significant effect of command type in Quiet (*F* = 7.676, *p* = 0.018) but not in Sound (*F* = 4.028, *p* = 0.070). As expected, the effect of trial was highly significant in both cases (*F* = 22.413, *p* < 0.001 and *F* = 8.480, *p* < 0.001, respectively).

Collectively, these analyses indicate that participants navigated more slowly when using the tactile belt than the auditory device, particularly in the Quiet conditions.

### Auditory Navigation Was More Compromised by the Introduction of Background Street Noise

Figure 4A indicates that participants’ performance was disrupted (i.e., completion time jumped upward) with the introduction of ambient noise (see dotted line connecting trials 5 and 6). Interestingly, the data suggest that ambient noise adversely affected navigation with the auditory device more than it did navigation with the tactile device. Two *post hoc* pairwise comparisons confirmed this impression. The increase in completion time from Auditory trial 5 (*M* = 46.68, *SD* = 11.52) to Auditory trial 6 (*M* = 53.96, *SD* = 11.88) was highly significant (*t*_(11)_ = 8.82, *p* < 0.001), whereas the increase in completion time from Tactile trial 5 (*M* = 52.21, *SD* = 12.03) to Tactile trial 6 (*M* = 55.98, *SD* = 12.22) was only marginally significant (*t*_(11)_ = 2.18, *p* = 0.052).

Additionally, Figure 4A suggests that completion times under Auditory commands—but not Tactile commands—remained compromised after the introduction of ambient noise, even with repeated testing over five trials. This was confirmed with two *post hoc* pairwise comparisons. The increase in completion times from Auditory trial 5 (*M* = 46.68, *SD* = 11.52) to Auditory trial 10 (*M* = 50.20, *SD* = 12.80) was significant (*t*_(11)_ = 3.30, *p* = 0.007), whereas the difference in completion time from tactile trial 5 (*M* = 52.21, *SD* = 12.03) to tactile trial 10 (*M* = 52.59, *SD* = 12.41) was not significant (*t*_(11)_ = 0.25, *p* = 0.808).

To further investigate these trends, we replotted the data from Figure 4A after dividing each participant’s completion times by the completion time on the first trial of the corresponding command type (Figure 4B). The normalized completion times for the two navigational devices appear to follow very similar courses as participants improved with practice in the Quiet conditions, but the completion times appear to worsen more for the auditory device in the Sound conditions. This was confirmed using a 2 × 2 × 5 (command type × ambient noise × trial) three-way repeated-measures ANOVA on the normalized completion times. The ANOVA revealed a significant command type × ambient noise interaction (*F* = 9.978, *p* = 0.009) and a significant effect of trial (*F* = 32.300, *p* < 0.001) but no significant effect of command type (*F* = 2.210, *p* = 0.165) or ambient noise (*F* = 4.491, *p* = 0.058). To further investigate the command type × ambient noise interaction, we conducted two *post hoc* two-way (command type × trial) repeated-measures ANOVAs, one for Quiet and the other for Sound. In keeping with the visual impression provided by Figure 4B, these ANOVAs revealed no significant effect of command type in Quiet (*F* = 0.085, *p* = 0.776) but a significant effect of command type in Sound (*F* = 5.815, *p* = 0.035).

These results indicate that background street noise compromised participants’ ability to navigate with auditory commands more than it compromised their ability to navigate with tactile commands.

### Straightaway and Turn Speeds Under Auditory Navigation Were More Compromised by Background Street Noise

**Figure 5:**
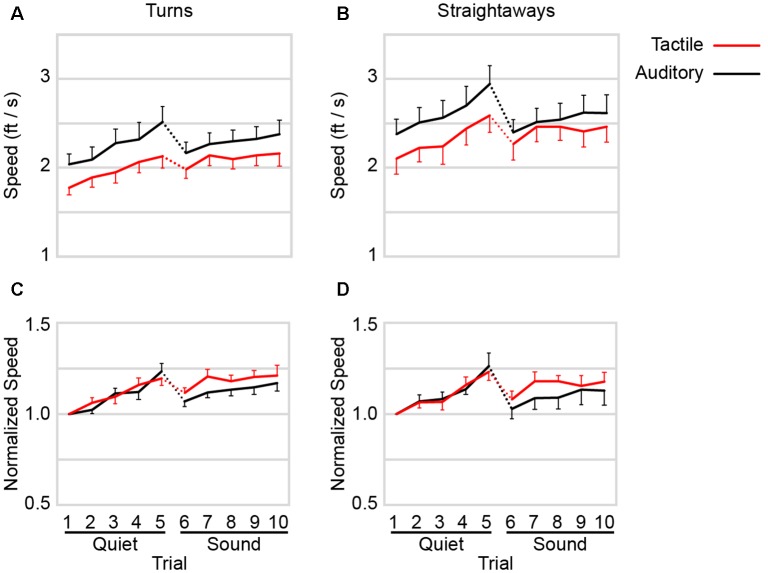
Navigational speed. **(A)** Mean turning speeds as a function of trial by command type. **(B)** Mean straightaway speeds as a function of trial by command type. **(C)** Mean normalized turning speeds. For each participant, turning speed on each trial was divided by the turning speed from trial 1 of the corresponding command type. **(D)** Mean normalized straightaway speeds. For each participant, straightaway speed on each trial was divided by the straightaway speed from trial 1 of the corresponding command type. Dotted lines: background street noise introduced to experiment. Red: tactile navigational commands. Black: auditory navigational commands. Error bars show +1 SE and −1 SE, respectively for the highest and lowest points at each comparison distance.

We next sought to determine how participants’ navigational behavior changed to account for the changes in completion times from Quiet to Sound observed in Figure 4. To this end, we focused on how participants adjusted their walking speed on the straightaway and turn components of the paths (Figure 5). Not surprisingly, the data indicate that participants tended to walk more rapidly on the straight portions of the path than they did when turning. Separate 2 × 2 × 5 (walking direction × ambient noise × trial) repeated-measures ANOVAs on the auditory and tactile navigation speeds revealed a highly significant main effect of walking direction (i.e., straight vs. turn) in both cases (auditory navigation: *F* = 33.794, *p* < 0.001; tactile navigation: *F* = 28.698, *p* < 0.001).

Both turning and straightaway speeds under auditory commands diminished by a larger magnitude—relative to tactile—with the introduction of background street noise (Figure 5). With the introduction of background street noise, turning speeds under auditory commands decreased (trial 5 to trial 6) from 2.52 ± 0.61 to 2.17 ± 0.41 ft/s, whereas turning speeds under tactile commands decreased from 2.13 ± 0.45 to 1.98 ± 0.36 ft/s (Figure 5A). Similarly, straightaway speeds under auditory commands decreased from 2.94 ± 0.71 to 2.40 ± 0.60 ft/s, whereas straightaway speeds under tactile commands decreased from 2.60 ± 0.71 to 2.27 ± 0.63 ft/s (Figure 5B). Thus, when participants were being guided by auditory commands, their walking slowed more noticeably with the introduction of background street noise.

These trends were confirmed by two 2 × 2 × 5 (command type × ambient noise × trial) three-way ANOVAs on turning and straightaway speeds. For turning speeds, the ANOVA revealed significant effects of command type (*F* = 16.276, *p* = 0.002), ambient noise (*F* = 7.107, *p* = 0.022), and trial (*F* = 22.248, *p* < 0.001), with a marginally significant command type × ambient noise interaction (*F* = 4.637, *p* = 0.054). Similarly, for straightaway speeds, the ANOVA revealed significant effects of command type (*F* = 14.143, *p* = 0.003) and trial (*F* = 22.105, *p* < 0.001), with a significant command type × ambient noise interaction (*F* = 5.757, *p* = 0.035); the effect of ambient noise on straightaway speeds was not significant (*F* = 0.008, *p* = 0.930).

To further investigate these trends, we replotted the data from Figures 5A,B after dividing each participant’s speeds by the speed on the first trial of the corresponding command type (Figures 5B,C). The normalized speeds for auditory and tactile navigation followed similar courses as participants improved with practice in the Quiet, but the speeds appeared to worsen more for the auditory device in Sound. Confirming this visual impression, a 2 × 2 × 5 repeated-measures ANOVA on the normalized turn speeds revealed a significant command type × ambient noise interaction (*F* = 5.084, *p* = 0.046). A 2 × 2 × 5 repeated-measures ANOVA on the normalized straightaway speeds revealed a non-significant interaction trend (*F* = 3.885, *p* = 0.072).

Collectively, these results suggest that ambient noise caused more interference with auditory than tactile navigation.

### The Participant Report Supported the Potential Usefulness of the Tactile Device

Results from the end-of-experiment questionnaire are displayed in Table 5. Participants responded strongly disagree, disagree, neutral, agree, or strongly agree to the following statements: (1) Overall, I think the belt would be helpful for navigation; (2) The signals given by the belt were clear and easy to feel; (3) I would find it easy to integrate the belt into my usual travel routines, using it in conjunction with my cane, guide dog or human guide; (4) The belt was comfortable to wear; and (5) I’d be better able to attend environmental sounds (traffic, someone talking, etc.) with the belt than with an audio navigation system. As indicated in the table, a clear majority of participants agreed or strongly agreed with these statements. Participants were particularly positive concerning the potential helpfulness of the belt for navigation (nine out of 12 participants—75%—strongly agreed, and the remaining three agreed). Ten of the 12 participants either agreed or strongly agreed that they would be better able to attend to environmental sounds when using the belt than an audio navigation system.

**Table 5:** Questionnaire responses.

	End-of-experiment questionnaire responses				
Statement	Response				
	Strongly disagree	Disagree	Neutral	Agree	Strongly agree
1	0	0	0	3 (25%)	9 (75%)
2	0	1 (8%)	2 (17%)	4 (33%)	5 (42%)
3	0	0	0	5 (42%)	7 (58%)
4	0	0	0	8 (67%)	4 (33%)
5	0	0	2 (17%)	4 (33%)	6 (50%)

## Discussion

The ability to travel independently is crucial to an individual’s quality of life but compromised by visual impairment. Several navigational aids have been developed for blind people to address this limitation. These devices typically employ auditory instructions to guide users to desired waypoints (Loomis et al., 2005; Gaunet, 2006). However, the use of auditory navigational commands may interfere with users’ awareness of their surroundings, with potentially detrimental consequences. There is an obvious need, then, to explore the use of alternative, under-utilized, sensory modalities to convey information for safe and independent travel. As spatial information can be readily conveyed to the skin and interpreted by the nervous system, tactile navigational aids would seem to hold particular promise. In the present study, we compared the efficacy of a novel tactile navigational aid and a conventional auditory aid. We predicted that: (1) in the absence of environmental sounds, navigation with the tactile aid would, with sufficient practice, be at least as good as navigation with the auditory aid; and (2) navigation with the tactile aid would be less impaired by concomitant attention to environmental sounds.

The data, while promising, offer a more nuanced view than we had envisaged. To our surprise, we found that, when participants navigated in a silent environment (Quiet conditions), they performed somewhat worse with the tactile belt than the auditory device, taking longer to complete each trial and committing more major errors. When participants navigated in the presence of simulated street noises (Sound conditions), the difference in completion time between auditory and tactile navigation diminished. These results suggest that tactile navigation, although not initially intuitive to the participants, holds promise as an effective navigational method in everyday environments characterized by ambient noise such as street sounds.

### Despite the Predicted Superiority of the Tactile Compared to the Auditory Modality for Navigational Processing, Participants Performed Worse When Using the Tactile Belt in the Quiet Conditions

Our findings support previous literature (Ertan et al., 1998; Tsukada and Yasumura, 2004; Van Erp et al., 2005; Pielot and Boll, 2010; Flores et al., 2015; Jimenez and Jimenez, 2017) revealing that tactile displays can successfully guide participants to waypoints. The present study also extends upon this body of literature, by comparing tactile to auditory navigational performance of blind participants in the presence or absence of street noise, and by providing participants with the opportunity to improve performance with repeated testing trials in every condition. Despite these differences in design, like Flores et al. (2015) and Jimenez and Jimenez (2017), we found that participants performed more slowly (in the Quiet conditions) when using the tactile belt than the auditory navigational device. Consistent with Jimenez and Jimenez (2017), we found also that participants made more errors in the tactile condition. These results contradicted our first prediction.

Spatial information is relayed from the skin through the central nervous system *via* topographically organized projections and is represented in somatotopic maps. In light of this spatial fidelity of the somatosensory system, it is not surprising that humans can extract spatial information from the positions of vibrotactile cues (Cholewiak et al., 2004; Van Erp, 2005; Jones and Ray, 2008). Accordingly, it would seem that the somatosensory system is ideally suited to extract navigational directions from vibrotactile stimulus patterns. By contrast, the perception of “turn left” or “turn right” commands requires acoustic, phonological and semantic processing across several separate brain regions, broadly distributed over the right and left hemispheres (Connolly and Phillips, 1994), suggesting that verbal commands require a greater degree of processing before their meaning can be extracted and translated into a spatial direction.

In light of these considerations, it surprised us that our participants generally performed better when using auditory commands than the tactile belt. This difference in performance may be attributed to the novelty of using a tactile device. Novel procedures often induce a cognitive load, resulting in diminished task performance (Sweller, 1994; Brunken et al., 2003; Haji et al., 2015). Participants in this study, who had little or no prior experience navigating with tactile commands, presumably had to process tactile instructions cognitively to a greater extent than an experienced user would have. In this vein, it is worth noting that several participants suggested that future versions of the tactile belt be accompanied by an intensity control to modulate the strength of vibration, as the participants had to expend effort to attend to the vibrations and sometimes missed them.

### Improvement With Practice Occurred at the Same Rate for the Two Command Types

Previous navigational studies (Pielot and Boll, 2010; Flores et al., 2015; Jimenez and Jimenez, 2017) did not investigate improvement with repeated testing. We did so by testing participants over five consecutive trials on the same path in each condition. We found that participants improved navigation at the same rate regardless of the command modality. A parsimonious explanation for this finding is that participants did not experience more difficulty acquiring information *via* one command modality than the other, such that the learning rate was constrained, not by the command type, but by the efficiency with which the participants were able to acquire a mental map of the spatial layout of the path.

As the participants repeatedly used the tactile belt, the intuitiveness of the tactile information presumably allowed them to build a spatial representation of the navigated path without excessive cognitive load (Brayda et al., 2013). The similar rates of improvement highlight the efficacy of the tactile belt in providing blind participants a spatial representation of their surroundings. These results suggest that, with practice, blind users could learn to efficiently navigate the real world with the tactile belt system.

### The Tactile Belt Benefited Users in the Presence of Background Street Noise

Unlike several previous tactile waypoint studies (but similar to Flores et al., 2015), our study simulated a realistic environment by adding ambient street noise to the navigational task (Sound conditions). Participants were asked to recall events from the street sounds as an incentive to actively attend to those sounds. This procedure simulated scenarios in which, for safety purposes, blind travelers must listen attentively to their immediate surroundings while navigating. We wondered how participants would fare while navigating with either the tactile belt or auditory device in the presence of high auditory load. We predicted that, when navigational instructions are processed through the tactile modality, the consequent mitigation of auditory load would result in two benefits: (1) superior navigational performance; and (2) better recall of the street sounds. Interestingly, although the results from our Sound conditions did not support these predictions, we found trends that strongly suggested the benefit of using tactile commands in an acoustically rich environment.

The primary findings from the Sound conditions would first seem to contradict our predictions, as the auditory performance was still marginally—but non-significantly—superior to tactile performance. However, as previously discussed, the results obtained from the present study were likely skewed to favor auditory commands due to the greater cognitive load associated with the novel tactile device. Hence, we considered it informative to investigate how performance changed from Quiet to Sound (Figure 4A). The results suggest that tactile navigational performance was less affected by high environmental auditory load. Specifically, tactile performance was less compromised with the introduction of background street noise as navigation completion times only slightly increased but subsequently improved to their previous levels by the end of the experiment. By contrast, the auditory performance was more compromised with the introduction of background street noise and failed to return to its previous levels. The normalized completion time data (Figure 4B) make apparent the extent to which auditory navigation was more compromised by background street noise, as do the normalized speed data (Figures 5C,D).

We consider two plausible explanations for why auditory navigation was more compromised by the introduction of background street noise. First, auditory commands and background street noises, when simultaneously present, may have interfered physically at the level of the acoustic waveform presented to the ears, such that in some cases the auditory command signal was physically corrupted or masked by a concomitant street sound. Second, even when physical interference did not occur, task performance may have been compromised in the complex acoustic environment consisting of both auditory commands and street noises, as the concurrent processing of two acoustic inputs may burden shared neural resources. In contrast, distinct sensory modalities may engage independent neural resources as well as shared ones (Wickens, 2008). Consequently, the processing of concurrent inputs—such as navigational commands and street sounds—may be achieved with less difficulty when the inputs are delivered through separate modalities (Duncan et al., 1997; Martens et al., 2010).

### Future Directions

The present study provides a proof of concept for a tactile navigational belt for blind individuals. The belt successfully guided users to waypoint destinations, while leaving the auditory modality to attend to environmental sounds. Although participant performance was somewhat better overall with the conventional auditory navigational device than with the novel tactile belt, the data show that performance with the belt improved upon repeated testing and suggest that navigation with the belt was less impaired by the presence of attention-demanding environmental sounds. These findings suggest that tactile navigation systems hold promise and should be further investigated and refined. In particular, future studies should be conducted in order to optimize stimulus timing and intensity, which can exert strong effects on spatial perception (Tong et al., 2016). More sophisticated tactile commands could be implemented, *via* the use of additional actuators, for instance, commands that include turn angles other than 90-degrees. In addition, long-term training should be conducted in order to measure participants’ asymptotic performance with the tactile navigational system. Previous research has shown that tactile acuity improves with training (Wong et al., 2013) and that blind individuals have the capacity for enhanced tactile processing (Bhattacharjee et al., 2010; Wong et al., 2011). Accordingly, we expect that, with sufficient practice, blind users would be able to integrate a tactile belt system seamlessly into their daily navigational activities. Ultimately, a tactile belt system may be combined with other advances, such as a technology-enhanced white cane (Khan et al., 2018), to form an integrated navigational assistance system.

## Data Availability Statement

The datasets generated for this study are available on request to the corresponding author.

## Ethics Statement

The studies involving human participants were reviewed and approved by McMaster University Research Ethics Board. The patients/participants provided their written informed consent to participate in this study.

## Author Contributions

SS was part of a team that conceptualized, designed, and built the tactile belt device. AB, SS and DG designed the study and wrote the manuscript. AB and SS conducted the experiments. AB and DG analyzed the data.

## Conflict of Interest

SS was a member of a team that designed and constructed the vibrotactile belt system used in this study. His team has founded a startup company that may market a tactile navigational device; the company is currently at the product verification stage.

The remaining authors declare that the research was conducted in the absence of any commercial or financial relationships that could be construed as a potential conflict of interest.
